# A Na^+^/K^+^ ATPase Pump Regulates Chondrocyte Differentiation and Bone Length Variation in Mice

**DOI:** 10.3389/fcell.2021.708384

**Published:** 2021-12-14

**Authors:** Marta Marchini, Mitchell R. Ashkin, Melina Bellini, Margaret Man-Ger Sun, Matthew Lloyd Workentine, Hamza Malik Okuyan, Roman Krawetz, Frank Beier, Campbell Rolian

**Affiliations:** ^1^ Department of Anatomy and Cell Biology, Cumming School of Medicine, University of Calgary, Calgary, AB, Canada; ^2^ McCaig Institute for Bone and Joint Health, University of Calgary, Calgary, AB, Canada; ^3^ Department of Comparative Biology and Experimental Medicine, Faculty of Veterinary Medicine, University of Calgary, Calgary, AB, Canada; ^4^ Department of Physiology and Pharmacology, Schulich School of Medicine and Dentistry, Western University, London, ON, Canada

**Keywords:** chondrocytes, Na^+^/K^+^ ATPase, bone length, Longshanks, growth plate, endochondral ossification, chondrogenesis

## Abstract

The genetic and developmental mechanisms involved in limb formation are relatively well documented, but how these mechanisms are modulated by changes in chondrocyte physiology to produce differences in limb bone length remains unclear. Here, we used high throughput RNA sequencing (RNAseq) to probe the developmental genetic basis of variation in limb bone length in Longshanks, a mouse model of experimental evolution. We find that increased tibia length in Longshanks is associated with altered expression of a few key endochondral ossification genes such as *Npr3, Dlk1, Sox9,* and *Sfrp1*, as well reduced expression of *Fxyd2,* a facultative subunit of the cell membrane-bound Na^+^/K^+^ ATPase pump (NKA). Next, using murine tibia and cell cultures, we show a dynamic role for NKA in chondrocyte differentiation and in bone length regulation. Specifically, we show that pharmacological inhibition of NKA disrupts chondrocyte differentiation, by upregulating expression of mesenchymal stem cell markers (*Prrx1, Serpina3n*), downregulation of chondrogenesis marker *Sox9*, and altered expression of extracellular matrix genes (e.g., collagens) associated with proliferative and hypertrophic chondrocytes. Together, Longshanks and *in vitro* data suggest a broader developmental and evolutionary role of NKA in regulating limb length diversity.

## Introduction

The size and shape of skeletal elements, particularly of the limbs, is a key driver of adaptive radiation in mammals (Smith and Savage, 1956). How variation in the size and shape of limb elements originates in embryogenesis has been a major focus of evolutionary developmental biology (Müller, 1991). During limb embryogenesis, mesenchymal stem cells (MSCs) initially form condensations in which the cells eventually differentiate into chondrocytes (Karsenty et al., 2009). These cartilaginous condensations (anlagen) are subsequently replaced by bone through the process of endochondral ossification (Mackie et al., 2008). Longitudinal growth of the skeletal anlagen occurs within the growth plate, a highly organized structure that contains a stem cell niche (resting zone) (Abad et al., 2002), a transit-amplifying zone (proliferative zone), and a differentiation zone in which cells undergo hypertrophy, producing and remodeling extracellular matrix (hypertrophic zone) (Mackie et al., 2008). In the distal growth plate, the terminal fate of hypertrophic chondrocytes remains unclear: some undergo programmed cell death, leaving a degraded matrix which is invaded by vasculature and bone-forming osteoblasts at the metaphysis, while several recent cell fate mapping studies show that a portion of these cells can also transdifferentiate into osteoblasts (Zhou et al., 2014; Cervantes-Diaz et al., 2017). Heritable length variation within and among bones is thought to be a product of variation in the initial anlagen size (Sears et al., 2006; Sanger et al., 2011), in the cellularity and mitotic activity of the proliferative zone (Kember, 1993; Rolian, 2008; Marchini and Rolian, 2018), and/or in the rate and extent of hypertrophy in individual hypertrophic cells (Cooper et al., 2013; Rolian, 2020).

Growth plate organization is conserved across mammals despite extensive diversity in bone size and proportions both within and among species (Schmidt and Fischer, 2009). Such a high degree of conservation in cellular aspects of growth plate structure indicates that selectable variation in limb bone size and shape likely has its roots in subtle differences in the genetic regulation of growth plate development and function.

Progression of chondrocytes through the growth plate is tightly regulated by several well-characterized signaling pathways and gene regulatory networks (Karsenty et al., 2009; Belluoccio et al., 2010; Kozhemyakina et al., 2015). Chondrocytes of the resting zone are characterized by strong expression of early mesenchymal fate markers such as *Prrx1* and *Sox9* as well as *PTHrP* (Mizuhashi et al., 2018; Matsushita et al., 2020). As these chondrocytes enter the proliferative zone, *Prrx1* and *PTHrP* expression is reduced, and chondrocytes begin to produce extracellular collagen II and aggrecan as they arrange into columns, parallel to the direction of elongation (Leung et al., 2011). Initiation of hypertrophy is accomplished through increased expression of *Runx2* and *Mef2C*, as well as downregulation of *Sox9*, and is accompanied by a change in extracellular matrix components, most notably a shift in the predominant forms of collagen from *Col2* to *Col10* (Kozhemyakina et al., 2015). Spatial organization of this expression is controlled by several major signaling pathways, including hedgehog signaling (Ohba, 2016; Haraguchi et al., 2019), bone morphogenetic protein (BMP) signaling (Nilsson et al., 2007; Garrison et al., 2017), and Notch signaling (Mead and Yutzey, 2009b; Zieba et al., 2020). Although it is well understood how this signaling controls chondrocyte state, it is less clear how transitions between states are induced and executed at a molecular and cellular level, and how this can be finely modulated to produce normal variation in growth plate activity.

To understand how variation in gene expression in the growth plate can produce heritable variation in bone length, we characterized the transcriptome of the proximal tibial growth plate in the Longshanks mouse. The Longshanks mouse consists of two mouse lines (LS1 and LS2) selectively bred in parallel for increased tibia length relative to body mass (Marchini et al., 2014). After 20 generations of artificial selection, each Longshanks line shows a 13–15% increase in tibia length compared to a random-bred Control line on the same genetic background (CD-1) while retaining identical body masses (Castro et al., 2019). This increase in limb bone length is observable at birth (Farooq et al., 2017) and appears to be accomplished primarily through an increase in the number of proliferative chondrocytes as well as a faster turnover of the hypertrophic zone (Marchini and Rolian, 2018). Here we show that both Longshanks lines’ increased tibia length is associated with a molecular pathway that modulates activity of the Na^+^/K^+^ ATPase pump (hereafter NKA pump) by decreasing expression of its facultative gamma subunit, *Fxyd2*. Using tissue and micromass culture, we further demonstrate that modulation of the NKA pump disrupts chondrocyte differentiation and tibia growth, revealing a novel and important role for the NKA pump in controlling progression of chondrocytes through the growth plate.

## Materials and Methods

### Animal Samples

The study was approved by the University of Calgary Health Sciences Animal Care Committee (protocols AC13-0077, AC17-0026). Mice were humanely euthanized and immediately processed for tissue collection as required by each protocol (see further). We used mice from different generations of the Longshanks artificial selection experiment (Marchini et al., 2014) for each experiment (see further). The artificial selection experiment consists of two lines of mice selectively bred for increased tibia length with respect to body mass (LS1 and LS2) and a Control group of random-bred mice from the same genetic background (CD-1) (Marchini et al., 2014).

### RNA Sequencing

Three LS1 and three Control mice from different families in generation F13 were used for RNA sequencing at 14 days old. After euthanasia, tibiae from both hindlimbs of several LS1 and Control mice were dissected, measured and weighed. Then, we collected the proximal epiphysis of each tibia (consisting of the growth plate, metaphysis, articular cartilage and secondary ossification center), which were snap-frozen in liquid nitrogen. The two tibial proximal epiphyses from each mouse were pooled for total RNA extraction using the RNeasy mini kit (Qiagen). Before total RNA extraction, the frozen epiphyses were homogenized using a ball mill (Braun Mikro-Dismembrator S) at 2600 rpm for 60 s. The samples were then processed and sequenced at the Center for Mouse Genomics at the University of Calgary. Total RNA-Seq kits (Life Technologies) were used to obtain Poly-adenylated mRNAs only from the total RNA and then converted into bar-coded cDNA libraries. The cDNAs were size selected using Agencourt AMPure XP beads and then amplified using polymerase chain reaction (PCR) to obtain around 50 ng of 240–270 base pairs length cDNAs. Equal amounts of each library were pooled together. Using the EZ-Bead system (Life Technologies) magnetic sequencing beads were added to the libraries. A yield of 2.2 billion beads was obtained. The samples were then sequenced on a 5500xl Genetic Analyzer (Life Technologies) to generate 35–50 base paired-end reads.

### RNAseq Data Processing and Analysis

The reads were mapped to the GRCm38 mouse reference genome (Ensembl Release 84) using HISAT2 v2.0.2 (Kim et al., 2015). In HISAT2, the options “–ss” and “–exon” were selected and read counts were created using the software featureCounts v1.4.6. Read counts were analyzed using DESeq2 (Love et al., 2014), as proxies for gene expression differences between Control and LS1. We considered different expression of a transcript to be significant when the *p*-value, adjusted for false discovery rates, was less than 0.05. To identify functional groups of differently expressed genes we used SPIA (Tarca et al., 2009) and the “g raphite” R package (Sales et al., 2012) together with the Reactome database (Croft et al., 2014). For additional details on the RNAseq see https://ucvm-bio.shinyapps.io/Longshanks_RNA-seq/.

### Growth Plate Quantitative RT-PCR

To validate our RNAseq results, we analyzed the gene expression of the total growth plate (resting, proliferative and hypertrophic zones) of LS1, LS2 and Control using quantitative reverse transcriptase polymerase chain reaction (hereafter qPCR). After euthanasia, fifteen pairs of tibiae from 14-day old mice from generation F24 were dissected in each line (*n* = 45 mice total) and placed in RNAlater (Qiagen).

We dissected the proximal tibial total growth plate from both tibiae under a Zeiss stereomicroscope, and then snap-froze the growth plates in liquid nitrogen. We pooled the growth plates from three mice (six total proximal growth plates) to produce five biological replicates in each line, and homogenized them using a ball mill (Braun Mikro-Dismembrator S) at 2600 rpm for 60 s. We then isolated total RNA using RNeasy mini kit (Qiagen). The total RNA was then reverse transcribed in cDNA using High Capacity cDNA Reverse Transcription Kit (Thermo Fisher Scientific). We added TaqMan Fast Advance Master Mix (Thermo Fisher Scientific) to the samples and qPCR analysis was performed using custom fast 96-well array plates (Thermo Fisher Scientific). The TaqMan assays used were: *Actb, C1qtnf3, Casp1, Col10a1, Col2a1, Ccnb1, Ccnd1, Dlk1, Efemp1, Epyc, Fas, Fgl2, Frzb, Fxyd2, Gdf10, Hapln1, Igf2, Ihh, Itga1, Matn3, Mest, Mmp13, Mpeg1, Ptgs2, Pthlh, Runx2, Serpina3n, Sfrp1, Sox9, Wnt5a*, *Anxa1, B2m, Bglap3, Bmp2, Casp3, Cox6b1, Ctsc, Gas5, Mb, Npr3, Parp9, Pdzk1ip1, Wisp3* (Supplementary Table S1). Genes were selected following an detailed literature review, in which we searched for genes of interest in combination with keywords including “chondrocyte,” “growth plate,” “cartilage,” “bone,” “cell proliferation,” “cell hypertrophy,” and “cell death.” The plates were read on QuantStudio 6 Flex Real-Time PCR System (Thermo Fisher Scientific) and cycle-to-threshold (CT) was calculated. For each 96 well plate containing 32 gene primers, we loaded one sample each for Control, LS1 and LS2. Three technical replicates were performed for the first two biological replicates. Because the technical replicates of the first two biological replicates were found to have standard deviation less than 0.25, for the other three biological replicates we performed only two technical replicates (standard deviation < 0.25). In total, we used five plates for the five biological replicates. Only one gene, *Bglap3*, had standard variation > 0.25 in all the plates’ wells, perhaps due to a manufacture defect of the custom 96-well plates. Outliers were identified using SPSS v25 and not considered in subsequent statistical analysis.

### Tibia Culture

We harvested embryos at embryonic stage E15.5 from timed pregnant CD1 females, and placed them in 1x phosphate buffered saline (PBS) on ice. The tibiae from a minimum of 14 embryos were dissected under a stereoscope (Olympus SZX12) and placed in culture media alpha-MEM with the addition of 0.05 g/L ascorbic acid, 0.108 g/ml β-glycerophosphate, 2 g/L bovine serum albumin, 1000 units/ml of pen-strep, 2.5 ml/L L-Glutamine (Agoston et al., 2007; Ulici et al., 2008). The media was changed every day for 6 days. The tibiae were cultured at 37°C and 5% CO_2_. For each embryo, one tibia was used as control and the contralateral element was used as experimental. The only difference between the control and the experimental bones was the daily addition of either ouabain octahydrate (Sigma) or monensin sodium salt hydrate (Sigma) in the media from the second day of culture. We used three different concentrations of ouabain, 100 µM (*n* = 5), 500 µM (*n* = 5) and 1 mM (*n* = 4), and their respective controls (*n* = 14 pooled), and three concentrations of monensin, 0.1 µM (*n* = 6), 1 µM (*n* = 5) and 10 µM (*n* = 6), and their respective controls (*n* = 17 pooled). After 6 days in culture the tibiae were removed from culture. Specimens were digitally photographed with a scale at day 0 and at day 6 under an Olympus SZX12 stereomicroscope using a camera SPOT Insight 2 and pictures were acquired with SPOT imaging software 5.1. We then took linear measurements of total tibia length using TPSdig2 (Rohlf, 2005). We then processed the tibia for histology or qPCR.

### Tibia Histology

At the end of the culture period, E15.5 tibiae were selected from control media and from treatments that had the greatest effect on longitudinal growth (1000 µM ouabain, 0.1 µM monensin), fixed in 10% NBF and decalcified using Cal-Ex IITM (Fisher Chemical). Tissues were then dehydrated, embedded in paraffin, and sectioned in the coronal plane at 4 µm. Sections were deparaffinized in xylene and hydrated. Tibia sections were stained using Wiegert’s Iron Haematoxylin (Sigma), 0.05% Fast-Green (FCF) (Sigma) and 0.1% Safranin-o solution (Sigma). The stained sections were imaged using a digital microscope (Axioplan 2, Zeiss) with attached camera (Optronics), using StereoInvestigator v7 or PictureFrame.

### Immunohistochemistry

Immunohistochemistry staining was performed as previously described (Marchini et al., 2019). Briefly, sections of embryonic tibiae that were treated with the highest concentrations of ouabain (500 and 1000 µM) were deparaffinized, treated with hydrogen peroxide (15 min, 3% in methanol), followed by membrane permeabilization and antigen retrieval (30 min, 0.1% Triton-X). Sections were then blocked with normal serum for an hour at room temperature and incubated overnight at 4°C with primary antibody (COLII 1:100, ab34712, AbCam; COLX 1:50, ab49945, AbCam; DLK1 1:50, 10636-1-AP, ProteinTech; FXYD2 1:20, 11198-1-AP, ProteinTech; KI67 1:100, ab15580, AbCam; NPR3 1:100, ab37617, AbCam; PRRX1 1:100, NBP1-06067, Novus Biologicals, RUNX2 1:400, ab192256, AbCam; SFRP1; 1:100, ab4193, AbCam; SOX9 1:300, AF3075, Bio-Techne). After washes, sections were incubated with HRP-conjugated secondary antibodies (SOX9 and PRRX1: Bio-Techne HAF109, 1:100; FXYD2 and RUNX2: AbCam ab6721; 1:100, COLX: AbCam ab205719, 1:200, all other primary antibodies: Novus Biologicals NB7160, 1:200) for an hour. Liquid diaminobenzene (DAB) substrate kit (Invitrogen or DAKO K346711) was used to detect primary antibodies and methyl green was used as a counterstain. The stained sections were imaged using a digital microscope (Axioplan 2, Zeiss) with attached camera (Optronics), using StereoInvestigator v7 (MBF BioScience, Williston, VT).

### Micromass Culture

E11.5 mouse embryos were dissected from timed pregnancies (Control line females). Fore- and hind limb buds were dissected on ice in Puck’s Saline A (PSA), and mesenchymal cells were dissociated in dispase (Dispase II, Sigma Aldrich) in chick serum at 37°C in an orbital shaker for 30–45 min. Cells were then resuspended in medium containing 36% Dulbecco’s Modified Eagle’s Medium (DMEM), 53% Ham’s nutrient mixture F12, 10% fetal bovine serum (FBS), 0.5% L-glutamine and 0.5% streptomycin-penicillin, and plated at high density (2 × 10^7^ cells/mL) in 10 µl droplets in 6-well culture plates (7 droplets/plate), with daily media changes as previously described (Stanton et al., 2004; James et al., 2005a; Woods et al., 2005), On day 3, media was supplemented with β-glycerophosphate and ascorbic acid to achieve final concentrations of 1 and 0.25 mM, respectively. Serial dilutions of ouabain (ouabain octahydrate, Sigma-Aldrich) or control vehicle (ddH_2_O) were introduced at 6, 8 or 10 days to the medium to achieve final concentrations of 1 µM, 10 µM, 100 µM and 1 mM ouabain. These time points were chosen to determine the impact of NKA pump inhibition at different stages of chondrocyte differentiation. Micromasses were harvested at selected time points for RNA extraction or histology (6, 8, 10 and 12 days post-plating, day 15 for histology).

### Micromass Histology

Micromasses harvested for histology at day 15 were washed and fixed in 3.7% neutral buffered formalin (NBF). After washes in PBS, the micromasses were treated with 0.2 M hydrochloric acid (HCl) and stained as follows: 0.1% Alcian Blue in 95% ethyl alcohol (EtOH) for 6.5 h, washed in 70% EtOH, 0.5% KOH for ∼6 min, 1% Alizarin Red S in PBS at PH = 6.3 for 16 h, washed in 70% EtOH, 95% EtOH and 2.0 M HCl. After staining, micromasses were stored and imaged in glycerol on a digital microscope (AxioPlan 2, Zeiss) with attached camera (Optronics), using Stereo Investigator v7 (MBF BioScience, Williston, VT).

### Embryonic Tibia and Micromass Quantitative RT-PCR

On selected harvest days, micromasses (6–8, 8–10, 10–12 and 6–12 days) and embryonic tibiae (day 6) were washed in PBS, and stored in Trizol at −80°C for further processing. Tissues were homogenized using 1.4 mm ceramic beads (VWR) with a Mini Bead Mill Homogenizer (VWR). Tissue in Trizol was then added to a Phasemaker tube (Thermo Fisher Scientific) following manufacturer’s protocol. Supernatant was removed and an equal volume of 70% ethanol was added. This was then added to RNEasy mini kit column (Qiagen) following the manufacturer’s protocol. The total RNA was then reverse transcribed in cDNA using High-Capacity cDNA Reverse Transcription Kit (Thermo Fisher Scientific). We used TaqMan Fast Advance Master Mix (Thermo Fisher Scientific). In the tibia cultures, four tibiae from each concentration of ouabain and monensin, including controls, were used. Tibiae qPCR was performed using custom made fast 96-well plates with primers for 16 genes (Thermo Fisher Scientific). The 16 TaqMan assays were: *18S, Actb, Bmp2, Casp3, Col2, Col10, Dlk1, Efemp1, Fasl, Fxyd2, Gdf5, Ihh, Prrx1, Serpina3n, Sox9,* and *Wnt5a. Fasl* did not amplify in most of the runs and was therefore excluded from further analyses. For micromass culture, four wells per concentration of ouabain (each containing 7 masses) were used as biological replicates. qPCR was performed using 384-well and 96-well plate using the following TaqMan assays (Thermo Fisher Scientific): *Actb, Acan, Adamts5, Bcl2, Col2, Col10, Ihh, Mapk14, Mmp13, Runx2, Sox9.* The plates were used on QuantStudio 6 Flex Real-Time PCR System (Thermo Fisher Scientific) and cycle-to-threshold (CT) was calculated.

### Statistical Analyses

For growth plates, embryonic tibiae, and micromass qPCR, we first calculated the mean cycle-to-threshold (CT) of the technical replicates for each biological replicate. When we found high standard deviation (>0.5) in three technical replicates, we excluded the replicate driving the high deviation. Mean CT was then normalized using the CT of *Actb* expression in the same samples, as a housekeeping gene. Significance of differences in normalized gene expression (deltaCT values) in the growth plates of Control, LS1 and LS2, and in the E15.5 tibia cultures, was determined using ANOVA, followed by Bonferroni post-hoc test to evaluate pairwise differences in expression. For micromasses, significance of any differences in gene expression (expressed as deltaCT) was determined using linear mixed models (LMMs). We used a full factorial LMM with time point and treatment concentrations (ouabain or monensin) as categorical factors, and biological replicate as a random factor. Significance of pairwise post-hoc differences in individual gene expression in micromass assays was determined using Tukey’s honestly significant differences (HSD). All statistical analyses were performed using SPSS v25 (IBM Corp. Armonk, NY: IBM Corp), Matlab R2020a (Natick, MA.) or Statistica v.10 (StatSoft, Tulsa, OK), and were considered significant at *p* < 0.05.

## Results

### Differential Gene Expression Between Longshanks and Control Using RNA Sequencing and qPCR

To identify transcriptional differences associated with the Longshanks phenotype, we compared the gene expression profile of the proximal tibia between random-bred Control and the selectively bred LS1 at 14 days postnatal. Gene expression data were generated using bulk transcriptomic sequencing (RNAseq, see *Material and Methods* for details). A total of 19,354 genes and their transcript abundance were mapped using HISAT2(Kim et al., 2015). We then compared the read counts between Control and LS1 using DESeq2 (Love et al., 2014). We found 421 genes that were differentially expressed between Control and LS1 (adjusted *p*-value *p* < 0.05) (Supplementary Table S2). An interactive database for comparing expression of all genes among Longshanks 1 and Control growth plate samples is available at the following website: https://ucvm-bio.shinyapps.io/Longshanks_RNA-seq/.

From our initial list of 421 differentially expressed genes, we then identified and selected 42 genes with both known and unknown roles in endochondral ossification for validation with qPCR using complete growth plate tissue, based on a literature review (see *Materials and Methods* section for details) (Table 1). This allowed us to compare broadly between LS1 and LS2, and limit our comparisons to differences in gene expression to the growth plate alone, rather than including tissues of the bone front, epiphysis, and joint surface included in the transcriptomic analysis. We used *Actb* as our reference gene to calculate the difference in PCR cycles-to-thresholds (ΔCT) and relative quantification (fold-change) (James et al., 2005b; Stephens et al., 2011).

**TABLE 1:** Genes validated using qPCR.

Symbol	Description	RNAseq and qPCR			Function
**Genes from RNAseq analysis with known function(s) in the growth plate**					
*Hapln1*	Hyaluronan and proteoglycans link protein 1	↑	LS1	1.0 (0.9–1.2)	Constituent of extracellular matrix (ECM) in resting zone
			LS2	1.1 (0.9–1.2)	
*Igf2*	Insulin like growth factor 2	↑	LS1	1.2 (1.0–1.5)	Growth factor expressed in proliferating chondrocytes
			LS2	1.2 (1.0–1.5)	
*C1qtnf3*	C1q and tumor necrosis factor Related Protein 3	↑	LS1	1.1 (0.8–1.5)	Regulates proliferation and migration of proliferative chondrocytes
			LS2	1.2 (0.8–1.7)	
*Dlk1*	Delta like non-canonical notch ligand 1	↑	LS1	1.7* (1.3–2.1)	Regulates proliferation and differentiation of proliferative chondrocytes
			LS2	2.0* (1.5–2.6)	
*Epyc*	Epiphycan	↑	LS1	1.0 (0.9–1.2)	ECM constituent involved in cartilage development and maintenance
			LS2	1.1 (0.9–1.4)	
*Frzb*	Frizzled-related protein	↑	LS1	0.8 (0.6–1.0)	Regulates chondrocyte maturation
			LS2	1.0 (0.8–1.4)	
*Sfrp1*	Secreted frizzled related protein 1	↑	LS1	1.2 (1.0–1.4)	Wnt antagonist, inhibits hypertrophic differentiation
			LS2	1.3* (1.1–1.6)	
*Fgl2*	Fibrinogen like 2	↑	LS1	1.1 (0.8–1.4)	Hypertrophic marker
			LS2	1.3 (1.0–1.7)	
*Matn3*	Matrilin 3	↑	LS1	1.1 (0.8–1.4)	ECM protein constituent
			LS2	1.1 (0.8–1.6)	
*Npr3*	Natriuretic peptide receptor 3	↑	LS1	0.5* (0.4–0.6)	Delays chondrocyte hypertrophy
			LS2	0.6* (0.4–0.8)	
*Wisp3*	WNT1 inducible signalling pathway 3	↑	LS1	1.0 (0.7–1.3)	Inhibits chondrocyte hypertrophy
			LS2	1.2 (0.9–1.7)	
*Bglap3*	Bone gamma-carboxyglutamate protein 3	↓	LS1	0.3* (0.2–0.4)	Osteoblast hormone, regulates bone metabolism and energy homeostasis
			LS2	**0.5° (0.3–0.9)**	
*Gdf10*	Growth differentiation factor 10	↑	LS1	1.3 (1.1–1.5)	Inhibits BMP signaling in the proliferative zone
			LS2	1.1 (0.9–1.4)	
*Runx2*	Runt related transcription factor 2	↑	LS1	0.9 (0.7–1.1)	Promotes hypertrophic chondrocyte differentiation
			LS2	1.2 (1.0–1.4)	
*Wnt5a*	Wnt family member 5a	↑	LS1	0.9 (0.8–1.1)	Initiates chondrocyte hypertrophy
			LS2	**0.8 (0.7–0.9)**	
*Col10a1*	Collagen type X alpha 1 chain	↑	LS1	0.8 (0.5–1.2)	Cartilage ECM constituent, expressed in hypertrophic zone
			LS2	0.9 (0.5–1.6)	
*Mest*	Mesoderm specific transcript	↑	LS1	0.9 (0.8–1.1)	Regulates proliferation and differentiation of proliferative chondrocytes
			LS2	1.1 (0.9–1.5)	
**Genes from RNAseq analysis with unknown function(s) in the growth plate**					
*Fas*	Fas cell surface death receptor	↑	LS1	1.3 (0.8–2.2)	Promotes programmed cell death
			LS2	1.5 (1.0–2.4)	
*Anxa1*	Annexin A1	↑	LS1	1.3 (0.9–1.8)	Promotes programmed cell death
			LS2	1.2 (0.7–1.8)	
*B2m*	Beta-2-microglobulin	↑	LS1	0.9 (0.7–1.0)	Promotes osteoclastogenesis
			LS2	0.9 (0.8–0.9)	
*Fxyd2*	FXYD domain containing ion transport regulator 2	↓	LS1	0.4* (0.3–0.5)	Regulates efficiency of the Na^+^/K^+^ ATPase pump
			LS2	0.3* (0.3–0.4)	
*Mb*	Myoglobin	↓	LS1	1.1 (0.8–1.5)	Promotes programmed cell death
			LS2	1.0 (0.7–1.4)	
*Efemp1*	EGF containing fibulin like extracellular matrix protein 1	↑	LS1	1.1 (0.8–1.4)	ECM constituent in resting zone
			LS2	1.2 (1.0–1.5)	
*Itga1*	Integrin subunit alpha 1	↑	LS1	0.9 (0.7–1.1)	Regulates mesenchymal stem cell proliferation
			LS2	0.9 (0.8–1.1)	
*Ptgs2*	Prostaglandin-endoperoxide synthase 2	↑	LS1	0.9 (0.6–1.3)	Inhibits cell proliferation and induces programmed cell death
			LS2	0.7 (0.5–0.9)	
*Casp1*	Caspase 1	↑	LS1	1.0 (0.6–1.5)	Promotes programmed cell death
			LS2	0.7 (0.5–1.0)	
*Mpeg1*	Macrophage expressed 1	↑	LS1	1.4 (0.6–3.6)	Marker of activated macrophages involved in immune response to bacterial infection
			LS2	0.6 (0.5–0.8)	
*Serpina3n*	Serpin family A member 3n	↑	LS1	1.2 (0.9–1.5)	Regulates cell growth and apoptosis
			LS2	1.3 (1.1–1.5)	
*Parp9*	Poly (ADP-Ribose) polymerase family member 9	↑	LS1	1.1 (1.0–1.2)	Promotes macrophage activation
			LS2	**1.2 (1.0–1.4)**	
*Gas5*	Growth arrest specific 5	↓	LS1	**0.6° (0.4–1.0)**	Promotes programmed cell death
			LS2	0.8 (0.6–1.1)	
*Pdzk1ip1*	PDZK1 interacting protein 1	↓	LS1	1.0 (0.8–1.3)	Inhibits programmed cell death
			LS2	1.4 (1.0–1.9)	
*Cox6b1*	Cytochrome C oxidase subunit 6B1	↓	LS1	1.0 (0.6–1.5)	Involved in mitochondrial respiration
			LS2	1.0 (0.6–1.6)	
*Ctsc*	Cathepsin C	↑	LS1	1.1 (0.9–1.4)	Induces programmed cell death
			LS2	1.1 (0.9–1.3)	
**Genes not differentially expressed from RNAseq analysis**					
*Sox9*	SRY-box 9	=	LS1	**0.7° (0.5–1)**	Delays chondrocyte hypertrophy
			LS2	0.6* (0.5–0.7)	
*Col2a1*	Collagen type II alpha 1 chain	=	LS1	0.9 (0.6–1.4)	Cartilage ECM constituent in proliferative zone
			LS2	0.9 (0.5–1.7)	
*Ihh*	Indian hedgehog	=	LS1	0.9 (0.6–1.4)	Stimulates chondrocyte proliferation
			LS2	1.0 (0.6–1.6)	
*Mmp13*	Matrix metallopeptidase 13	=	LS1	0.9 (0.7–1.1)	ECM proteinase expressed in hypertrophic zone
			LS2	0.9 (0.7–1.1)	
*Bmp2*	Bone morphogenetic protein 2	=	LS1	0.9 (0.7–1.0)	Stimulates chondrocytes hypertrophy
			LS2	0.9 (0.8–0.9)	
*Pthlp*	Parathyroid hormone-like hormone	=	LS1	1.1 (1.0–1.4)	Inhibits chondrocyte hypertrophy
			LS2	1.3 (1.0–1.7)	
*Casp3*	Caspase 3	=	LS1	1.0 (0.6–1.6)	Promotes programmed cell death
			LS2	1.2 (0.7–1.9)	
*Ccnd1*	Cyclin D1	=	LS1	0.8 (0.6–1)	Increases proliferation through the G1 phase
			LS2	0.8 (0.6–0.9)	
*Ccnb1*	Cyclin B1	=	LS1	1.3 (1.0–1.7)	Cell proliferation: G2/M phase marker
			LS2	1.1 (0.9–1.2)	

↑ indicates upregulation in RNAseq.

↓ indicates downregulation in RNAseq method.

= indicates no difference in expression.

LS1 indicates Longshanks Line 1 (*n* = 5 biological replicates).

LS2 indicates Longshanks Line 2 (*n* = 5 biological replicates).

The number after LS1 and LS2 indicates the fold change from qPCR results using the delta-deltaCT method, with 95% confidence intervals estimated from the difference in means and standard deviations of deltaCT in Longshanks and Controls. Bold font and asterisk indicate statistically significant differences from the Control line (*n* = 5 biological replicates) mean at *p* < 0.05, while bold font and circle indicates *p*-value *p* < 0.1 (ANOVA on deltaCT values, Bonferroni post-hoc correction).

Only a few genes were differentially expressed in the Longshanks growth plate (*Fxyd2, Dlk1, Npr3, Bglap3, Sfrp1* and *Sox9*) (Table 1, Supplementary Table S3). Of these, only *Fxyd2*, *Dlk1*, and *Npr3* are differentially expressed in both Longshanks lines. Significant differences in *Bglap3* (osteocalcin)*, Sfrp1* and *Sox9* are found in only one of the two lines, although in each case the other line shows a similar directionality in the change in mean expression relative to Controls. The greatest observed difference in relative quantification (fold-change) was downregulation of expression in *Fxyd2*, which was estimated to be 0.4 times as high in LS1 and 0.3 in LS2 relative to Controls (Table 1). *Fxyd2* is a facultative gamma subunit of the Na^+^/K^+^ ATPase pump (NKA pump) which modulates cation affinity of the pump (Geering, 2008), but a role in endochondral ossification has not previously been described.

Immunohistochemical analyses in proximal tibia epiphyses indicate that FXYD2 protein is localized primarily to the proliferative zone and articular cartilage in a spatial pattern that largely overlaps with expression of the chondrogenic transcription factor SOX9, (Supplementary Figure S1). *Sox9* is also significantly downregulated in LS2, with a similar trend observed in LS1. Immunostaining against SOX9 and FXYD2 appears to be weaker and more sparse in Longshanks vs. Control growth plates (Supplementary Figures S2A,B) (Agoston et al., 2007). *Dlk1* is upregulated, whereas *Npr3* is downregulated in both LS lines. Immunostaining against NPR3 suggests that this protein is less abundant in the proliferative zone in Longshanks (Supplementary Figure S2C), while DLK1 shows more intense staining in the resting zone, and in the proliferative zone including potentially its ECM (Supplementary Figure S2D). Finally, *Sfrp1* is upregulated in LS2, with a similar trend in LS1, however immunostaining against SFRP1 did not show any substantive differences in abundance between Control and Longshanks (Supplementary Figure S2E).

### Inhibition of the NKA Pump Affects Bone Growth and Chondrocyte Differentiation in Tibia Organ Cultures

**FIGURE 1:**
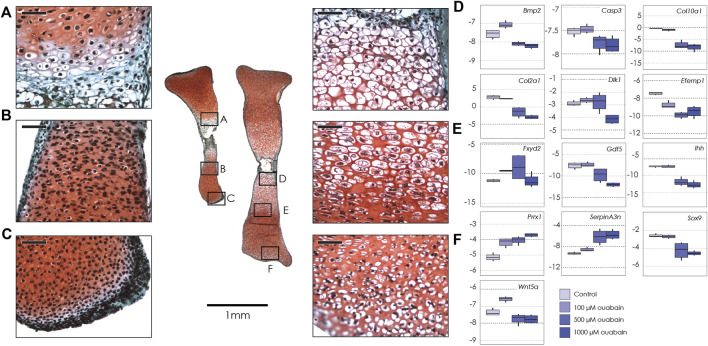
Tibiae cultured in ouabain octahydrate. Left: sections of two tibiae from a single E15.5 mouse fetus cultured in 1 mM ouabain **(left)** and control media **(right)**, stained with fast green and safranin-o. Black boxes show magnification of hypertrophic chondrocytes (**A**, 1 mM ouabain; D, control); proliferative and pre-hypertrophic chondrocytes (**B**, 1 mM ouabain; **E**, control) and epiphyseal/articular cartilage (**C**, 1 mM ouabain; **F**, control) Scale bar for magnified areas = 50 µm. Right: Boxplots of negative ΔCT for each gene (*y*-axis, where increasingly negative values represent lower expression levels relative to the housekeeping gene *Actb*), tested at different concentrations of ouabain octahydrate (0 µM or control, 100 µM, 500 µM and 1 mM). Sample size *n* = 4 for all concentrations. For clarity, significant differences between ouabain and vehicle concentrations within gene are not shown, but are reported in Table 2.

Given the marked differential expression of the facultative NKA subunit *Fxyd2* in our analysis of transcription in the Longshanks growth plate, we asked whether alterations in NKA function would impact cellular mechanisms of endochondral ossification. Specifically, we hypothesized that changing the functionality of the NKA pump would affect differentiation and/or relevant physiological processes such as cell swelling/hypertrophy, in growth plate chondrocytes. To test this, we cultured tibiae from E15.5 embryos, as well as limb bud micromasses (see below), in the presence of the cardiac glycoside ouabain (Nguyen et al., 2007; Sandtner et al., 2011), a known pharmacological inhibitor of the NKA pump. Tibiae treated with 100 µM ouabain and control media increased in length by 40–50% over the 6 days, whereas the tibiae treated with 1000 and 500 µM ouabain increased in length by only ∼15–16% (Figure 1, Supplementary Figure S3, Supplementary Table S4). Hence, NKA inhibition by ouabain was sufficient to suppress longitudinal growth of mouse fetal tibiae in culture.

Given the reduced longitudinal growth, we asked if, and how, treatment with ouabain had induced changes in the structure of the nascent growth plate. Histology showed that in tibiae treated with 1000 and 500 µM ouabain, the growth plate did not have a typical proliferative zone with flattened cell columns when compared to 100 µM ouabain and control media (Figure 1A). Moreover, proliferative and hypertused previously to counteractrophic chondrocytes were fewer in number in tibiae treated with 1000 µM ouabain (Figures 1A,D). In addition, in the region immediately distal to the hypertrophic cells, the chondrocytes were present at lower density, with smaller cytosolic spaces than in control tibiae. Similarly, cells in the distal epiphysis and articular cartilage of tibiae treated with 1000 µM are small and rounded, without the presence of cytosolic space observed in the chondrocytes in the same region in tibiae cultured in control media (Figures 1C,F). These data suggest that inhibition of the NKA pump with ouabain inhibits longitudinal growth, potentially by disrupting progression, differentiation and possibly hypertrophy of chondrocytes in the growth plate and articular cartilage.

As ouabain appears to interfere with chondrocyte proliferation, hypertrophy and/or differentiation, we hypothesized that disruption of the NKA pump’s activity might interfere with expression of genes with known function in the growth plate, including genes that are differentially expressed in Longshanks (Table 1). We collected RNA from whole tibiae cultured in different concentrations of ouabain and quantified the expression of key genes related to chondrogenesis and growth plate function by qPCR (Table 2 and Supplementary Table S5). We found that ouabain affects the expression of chondrogenesis genes in a dose-dependent manner. Culture of tibiae in high concentrations of ouabain increased expression of the MSC marker *Prrx1* (ten Berge et al., 1998), although its expression is more sensitive to ouabain and increases even at a concentration of 100 µM. Conversely, proliferative (*Sox9, Dlk1, Col2a1*), pre-hypertrophy (Ihh), and hypertrophy markers (*Bmp2, Col10a1, Gdf5*) were downregulated (Coleman and Tuan, 2003; Harkness et al., 2009; Shu et al., 2011; Usami et al., 2016).

**TABLE 2:** Gene expression in tibia culture using qPCR.

Symbol	Gene name	Ouabain FC	Monensin FC	Function
*Bmp2*	Bone morphogenetic protein2	**1.41 (1.15–1.73)**	0.87 (0.59–1.28)	Stimulates chondrocyte hypertrophy
		**0.69 (0.58–0.83)**	***0.47 (0.34–0.65)***	
		**0.64 (0.53–0.77)**	1.25 (0.81–1.93)	
*Casp3*	Caspase 3	1.03 (0.95–1.12)	1.07 (0.79–1.45)	Promotes programmed cell death
		0.84 (0.73–0.97)	*0.53 (0.38–0.74)*	
		0.81 (0.72–0.92)	*0.55 (0.41–0.74)*	
*Col10a1*	Collagen type X alpha 1 chain	0.64 (0.47–0.88)	***0.05 (0.01–0.28)***	Major cartilage ECM protein, preferentially expressed in hypertrophic zone
		***0.007 (0.002–0.021)***	***0.001 (0.001–0.001)***	
		***0.004 (0.001–0.011)***	***0.014 (0.003–0.06)***	
*Col2a1*	Collagen type II alpha 1 chain	0.75 (0.51–1.1)	0.53 (0.26–1.09)	Major cartilage ECM protein, preferentially expressed in proliferative zone
		***0.05 (0.02–0.15)***	***0.002 (0.001–0.003)***	
		***0.02 (0.01–0.03)***	***0.06 (0.02–0.17)***	
*Dlk1*	Delta like non-canonical notch ligand 1	1.2 (1–1.43)	0.73 (0.52–1.03)	Regulates proliferation and differentiation of proliferative chondrocytes
		1.09 (0.66–1.8)	***0.02 (0.01–0.04)***	
		***0.42 (0.32–0.56)***	***0.22 (0.14–0.32)***	
*Efemp1*	EGF containing fibulin like extracellular matrix protein 1	***0.4 (0.3–0.52)***	0.6 (0.34–1.05)	ECM constituent in resting zone and articular cartilage
		***0.18 (0.14–0.23)***	***0.13 (0.06–0.32)***	
		***0.24 (0.16–0.37)***	***0.06 (0.02–0.19)***	
*Fxyd2*	FXYD domain containing ion transport regulator 2	***3.21 (2.73–3.76)***	***2.74 (1.5–5)***	Modulates efficiency of the Na^+^/K^+^ ATPase pump
		***4.87 (1.06–22.4)***	***5.53 (3.23–9.46)***	
		0.97 (0.44–2.11)	***5.39 (2.96–9.84)***	
*Gdf5*	Growth differentiation factor 5	1.16 (0.65–2.07)	**2.53 (0.53–12.14)**	Regulates condensation and differentiation of chondrogenic precursors
		***0.22 (0.07–0.73)***	***0.04 (0.01–0.14)***	
		***0.05 (0.03–0.08)***	***0.07 (0.02–0.28)***	
*Ihh*	Indian hedgehog	0.96 (0.71–1.29)	**0.25 (0.09–0.67)**	Stimulates chondrocyte proliferation
		**0.04 (0.02–0.09)**	***0.01 (0.01–0.02)***	
		**0.02 (0.01–0.04)**	***0.03 (0.01–0.1)***	
*Prrx1*	Paired related homeobox 1	*1.97 (1.54–2.52)*	1.44 (0.96–2.16)	Mesenchymal stem cell marker
		***2.16 (1.71–2.73)***	1.14 (0.91–1.42)	
		***2.71 (2.28–3.23)***	0.82 (0.6–1.11)	
*Serpina3n*	Serpin family A member 3	1.67 (1.27–2.19)	***14.84 (9.74–22.62)***	Regulates cell growth and apoptosis
		***9.09 (3.54–23.39)***	1.23 (0.89–1.7)	
		***11.95 (6.57–21.73)***	***0.31 (0.18–0.52)***	
*Sox9*	SRY-box 9	0.94 (0.78–1.12)	0.75 (0.44–1.27)	Inhibits chondrocyte hypertrophy
		***0.32 (0.16–0.61)***	***0.19 (0.13–0.27)***	
		***0.25 (0.21–0.31)***	**0.49 (0.32–0.75)**	
*Wnt5a*	Wnt family member 5a	*1.73 (1.47–2.04)*	0.9 (0.55–1.48)	Inhibits chondrocyte hypertrophy
		0.77 (0.6–0.99)	***0.29 (0.22–0.39)***	
		0.76 (0.61–0.94)	***0.18 (0.11–0.29)***	

Relative quantification or fold changes (FC) in tibiae treated with 100, 500 and 1000 µM ouabain (*n* = 4 each), and 0.1, 1 and 10 µM monensin (*n* = 4 each), listed in increasing order. Values are mean fold-change in relation to control tibiae, with 95% confidence intervals estimated from the difference in means and standard deviations of deltaCT in treatment and control. Bold font when there is a minimum of two-fold change (FC) (<0.5 downregulation, >2 upregulation compared to control). Italic font indicates statistically significant difference between treatment concentration and control media at *p* < 0.05 (ANOVA on deltaCT values, Bonferroni post-hoc).

Markers of cells from the articular cartilage of the joint surface (*Efemp1, Gdf5*) also appear to be downregulated, consistent with the undifferentiated appearance of the chondrocytes of the joint surface (Figures 1C,F) (Coleman and Tuan, 2003; Hasegawa et al., 2017). We also found an increase in expression of *SerpinA3n*. SerpinA3 protease inhibitors (anti-chymotrypsins) have been shown to be expressed in chondrocytes, where they may regulate both extracellular matrix turnover and cell differentiation (Boeuf et al., 2008). Ouabain at any concentration does not appear to increase expression of the cell death marker *Casp3* (Porter and Jänicke, 1999). Interestingly, expression of *Wnt5a* and *Bmp2* appears to be upregulated at low concentrations of ouabain, suggesting a dynamic transcriptional response to NKA pump inhibition. Expression of *Fxyd2* does not show clear directional change across treatments, suggesting that inhibition of the NKA pump does not control expression of this facultative protein.

Next, we validated the qPCR data with immunostaining against selected proteins. The most consistent differences in protein expression between control and ouabain-treated tibiae were seen in the low-density band of cells adjacent to the hypertrophic chondrocytes in the treated tibiae, and in their epiphyses (Supplementary Figure S4). In the low cell density zone, several proteins associated with both proliferation (KI67, SOX9, COLII, NPR3, SFRP1, DLK1 Supplementary Figures S4A–F) and hypertrophy (RUNX2, COLX, Supplementary Figures S4G,H) are present. Immunostaining patterns in this zone are most similar to the pre-hypertrophic zone in the control tibia, suggesting that the chondrocytes in this region may be transitional. Strong immunostaining against the mesenchymal stem cell marker PRRX1 in this zone in ouabain-treated tibiae further suggests that these cells maintain or perhaps regain some pluripotency (Supplementary Figure S4I). In the epiphyses of control tibiae, all tested proteins are detected except for RUNX2 (Supplementary Figure S4G). In contrast, only COLII, COLX and FXYD2 are detected in the ouabain-treated epiphyses (Supplementary Figures S4C,H,J). The smaller lacunae and absence of most tested proteins suggests the cells in the treated epiphyses are undifferentiated, or at least not as metabolically active as they are in control tibiae. While Collagens II and X proteins are detected in the treated epiphysis, the staining is reduced compared to Control tibiae, consistent with the observed qPCR data. FXYD2 protein is present in the lower hypertrophic zone and epiphysis in control tibiae, but only in the transitional cells in the treated tibia, and in its epiphysis (Supplementary Figure S4J).

To further elucidate the potential function of NKA pump activity in chondrocytes, we performed a complementary experiment in which we sought to stimulate the pump in tibia culture. Stimulation of the pump can be achieved indirectly through increasing Na^+^ intracellularly using the ionophorous antibiotic monensin (Hatou et al., 2010; Mendoza et al., 1980; Petersson et al., 2006; Phornphutkul et al., 2006; Mounier and Posner, 2006), which has been used previously to counteract the effects of ouabain (Picton et al., 2017).

**FIGURE 2:**
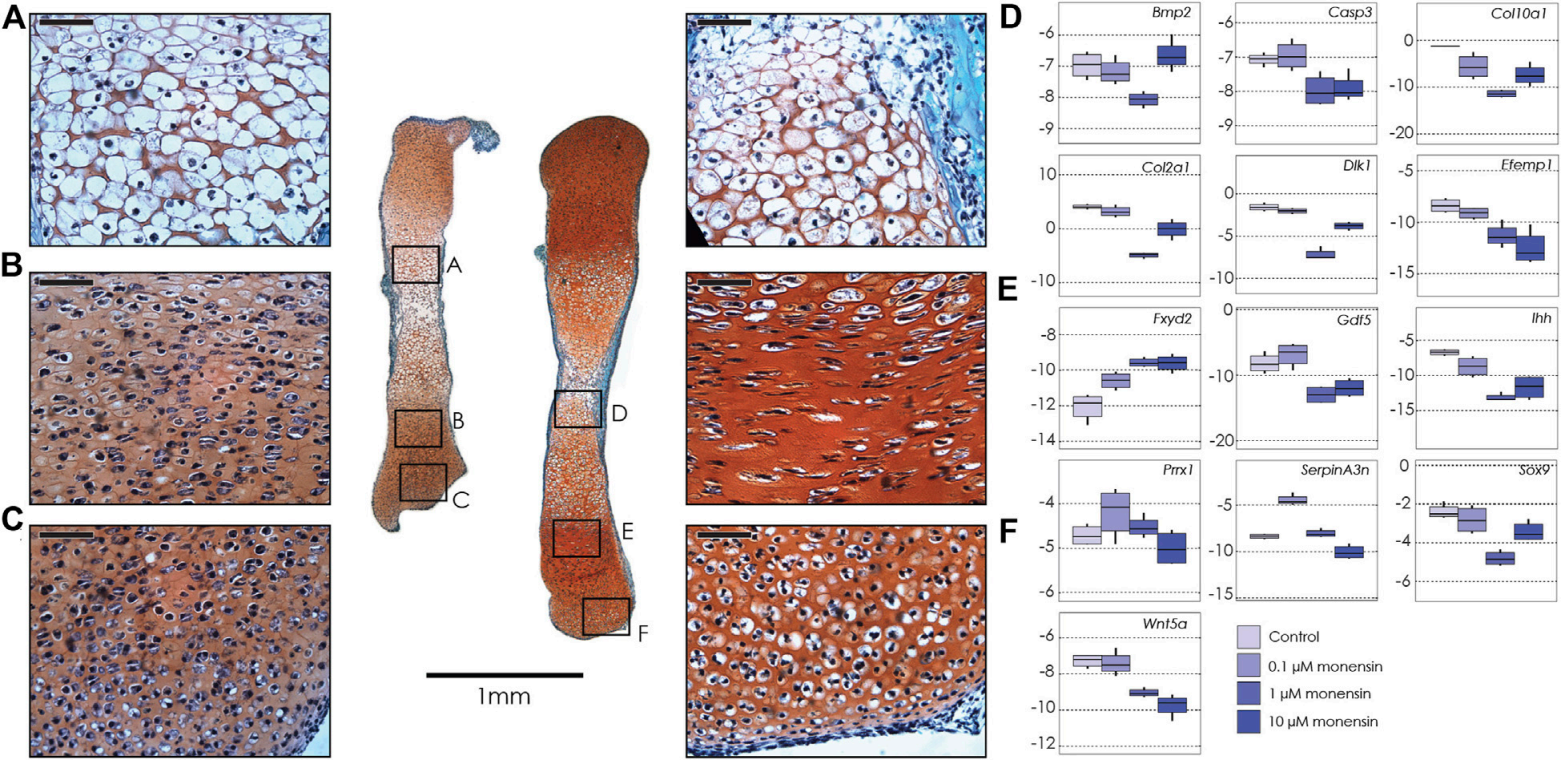
Tibiae cultured in monensin. Left: Sections of two tibiae from a single E15.5 mouse fetus cultured in 0.1 µM monensin **(left)** and control media **(right)**, stained with fast green and safranin-o. Black boxes show magnification of hypertrophic chondrocytes (**A**, 0.1 µM monensin; **D**, control); proliferative chondrocytes (**B**, 0.1 µM monensin; **E**, control) and articular cartilage (**C**, 0.1 µM monensin; **F**, control). Scale bar for magnified areas = 50 µm. Right: Boxplots of negative ΔCT for each gene (*y*-axis, where increasingly negative values represent lower expression levels relative to the housekeeping gene *Actb*), tested at different concentrations of monensin (0 µM or control, 0.1, 1 and 10 µM). Sample size *n* = 4 for all concentrations. For clarity, significant differences between ouabain and vehicle concentrations within gene are not shown, but are reported in Table 2.

We used three different concentrations of monensin: 0.1, 1, and 10 µM. As in the ouabain cultures, control tibiae increased in length by ∼40% over 6 days in culture. In contrast, all tibiae treated with monensin increased in length by ∼25%, irrespective of the monensin concentration, suggesting that there is no dose dependence of monensin on tibia growth response, at least not in the range of concentrations tested (0.1–10 µM) (Supplementary Figure S3, Supplementary Table S4). Histological sections show that tibiae cultured in monensin have a disorganized growth plate without a clear proliferative zone with flat cells organized into columns. In contrast, the articular cartilage and the hypertrophic zone are not as strongly affected (Figure 2).

As in the tibiae cultured with ouabain, we collected RNA from whole tibiae and used qPCR to quantify the expression of the same genes (Table 2, Supplementary Table S6). We found that, similarly to ouabain, monensin affects the expression of chondrogenesis genes. Unlike ouabain cultures, however, the relationship between monensin dose and gene expression appears to be less linear. For example, both *Col2a1* and *Col10a1* are downregulated, as well as proliferative markers (*Sox9, Dlk1*) and hypertrophy markers (*Bmp2, Gdf5*), but the effect of monensin on differential expression of these genes is greatest at an intermediate concentration (1 µM). Markers of cells from the articular cartilage of the joint surface (*Efemp1, Gdf5*) also appear to be downregulated along with *Wnt5a*, which promotes chondrocyte columnar organization and hypertrophic differentiation. As with ouabain, monensin does not appear to increase the cell death marker *Casp3*. There are a few notable differences in gene expression between tibiae cultured in ouabain and monensin: in the latter, *Fxyd2* is upregulated in all concentrations; *Serpina3n* is upregulated at low dosage while downregulated at higher concentrations of monensin; and *Prrx1* is not differentially expressed. These data suggest that activation of the NKA pump using monensin has an inhibitory effect on tibia growth, but this may not be mediated through mesenchymal cell differentiation.

### Inhibition of the NKA Pump Affects Chondrocyte Size and Differentiation in Micromass Culture

**FIGURE 3:**
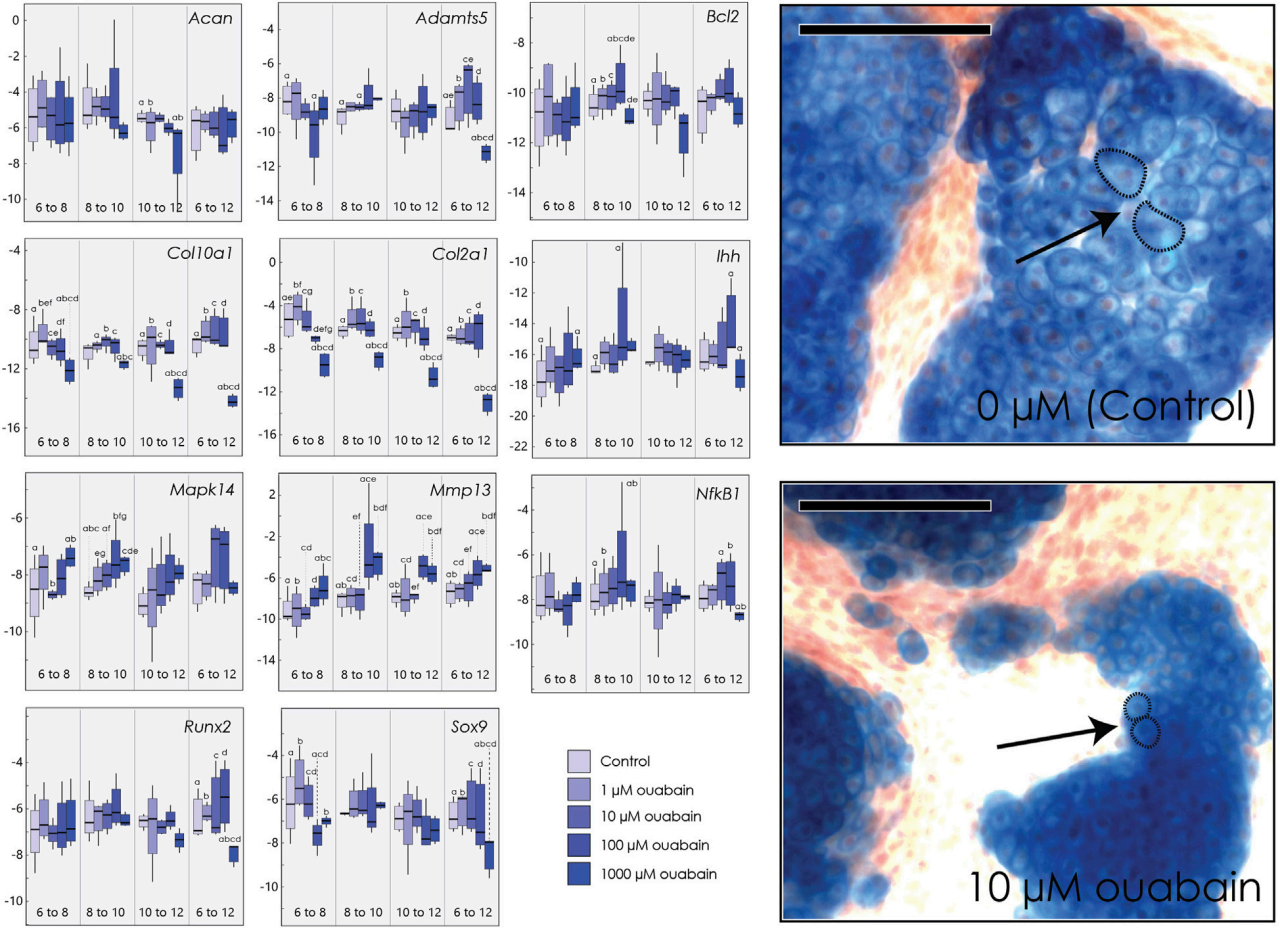
Micromasses cultured in ouabain octahydrate. Left: Boxplots of negative ΔCT for each gene (*y*-axis, where increasingly negative values represent lower expression levels relative to the housekeeping gene *Actb*) cultured and harvested at different day ranges (*x*-axis), and tested at different concentrations of ouabain (0 µM or control, 1, 10, 100 and 1000 µM). Sample size *n* = 4 for all concentrations. Superscripts denote significant pairwise differences among concentrations within a treatment duration, determined using Tukey’s HSD. Right: Micromasses stained with Alcian blue and alizarin red after 15 days in culture media with 0 µM ouabain **(control, top)** and with 10 µM ouabain **(bottom)**, introduced at day 6. Arrows indicate relative size of individual chondrocytes, outlined with a dashed line. Scale bar = 100 µm.

Because our data shows that ouabain appears to interfere with chondrocyte proliferation and hypertrophy in tibia culture, we hypothesized that the effects of inhibiting the NKA pump may be stage-dependent. To test this, we cultured micromasses derived from embryonic limb buds and introduced ouabain at different stages of chondrocytes differentiation, and at four different concentrations (1, 10, 100, 1000 µM) (Figure 3). We inhibited the NKA pump in micromasses by introducing ouabain to the culture medium at different days (Day 6, Day 8, Day 10). Micromasses were then harvested at different days (Day 8, Day 10, Day 12). This experimental design allowed us to inhibit the pump at initiation of chondrocyte proliferation (6–8 days), during proliferation (8–10 days), and at initiation of hypertrophy (10–12 days) (James et al., 2005b). We then extracted RNA, which we quantified using qPCR for several key genes involved in chondrocyte differentiation (Figure 3).

As in our tibia culture, we found that culturing cells in higher concentrations of ouabain decreases expression of *Col2a1*, when ouabain is introduced at any stage in differentiation (Supplementary Table S7). Similarly, high concentrations of ouabain decrease *Col10a1* expression in chondrocytes when hypertrophic chondrocytes are present (days 10–12). At early stages (day 6–8), high concentrations of ouabain appear to decrease expression of *Sox9* and *Col10a1*, although this is not uniformly statistically significant (Figure 3). The matrix-metalloproteinase *Mmp13*, which mediates extracellular matrix regulation of chondrocyte terminal differentiation (Kozhemyakina et al., 2015), is also upregulated in response to high ouabain concentrations beginning in early stages (day 6–8) and even more so at later stages of micromass differentiation (day 8–10 and day 10–12) (Figure 3). In contrast to embryonic tibia cultures, we did not find clear differences in the core matrix protein aggrecan (*Acan*), pre-hypertrophy marker *Ihh*, hypertrophic chondrocyte and osteoblast marker *Runx2*, the differentiation marker *Nf-kB1* (Jimi et al., 2019), or the matrix degradation marker *Adamts5* (Seuffert et al., 2018). As in tibial culture, we did not observe substantial changes in cell death markers, here *Bcl2* (Wang et al., 2006), but when present, the changes suggest a decrease in its expression at higher ouabain concentrations.

We further characterized the histological structure of micromass colonies after 15 days in culture, with ouabain introduced at day 6. While at 1 µM concentration there are no visible differences, micromasses cultured in 10 µM ouabain or greater are smaller, with fewer smaller and more sparsely distributed nodules. Moreover, chondrocytes cultured in higher concentrations of ouabain are smaller and more densely packed. We also observed that micromass nodules at higher concentrations of ouabain are surrounded by a denser calcified matrix as indicated by alizarin red staining (Supplementary Figure S5).

## Discussion

### Na^+^/K^+^ ATPase Pump in Endochondral Ossification

An unexpected finding from our comparative RNAseq analysis of Control and Longshanks tibiae was the strong and consistent downregulation of *Fxyd2* in the latter. *Fxyd2* is a gamma subunit of the NKA pump that modulates activity of the pump by changing its affinity for Na^+^ and K^+^ ions, and/or for ATP (de Baaij et al., 2015; Wang et al., 2015). The NKA pump transports 3 Na^+^ outside of the cell in exchange for 2 K^+^ using ATP (Castillo et al., 2015). This exchange of cations between the cell and interstitial fluid is important to maintain the osmotic stability and membrane polarity of the cells (Armstrong, 2003).

Previous work has shown that NKA pump subunits are highly expressed in chondrocytes (Mobasheri et al., 2012), indicating a potentially important role in chondrocyte physiology and/or differentiation, but to date that role has not been well characterized. One role of NKA in chondrocytes could be to regulate the process of cell swelling associated with hypertrophy, by regulating osmotic changes across the cell membrane. Our histological data lend some support to this hypothesis, as chondrocyte cell size appears to be reduced when the NKA pump is inhibited in both tibia and cell cultures (Figures 1, 3). We show here that inhibition of the pump with ouabain also disrupts gene expression associated with normal differentiation of chondrocytes. Specifically, well-established markers of chondrogenic precursors (*Prrx1, Serpina3n*) (Boeuf et al., 2008; Liu et al., 2017) are upregulated in tissues and cells cultured in ouabain, whereas markers of proliferative (*Col2a1, Ihh, Sox9*), prehypertrophic (*Ihh*), and hypertrophic (*Col10a1*) chondrocytes are downregulated in these same tissues.

Moreover, consistent and stage-dependent differential expression of collagen matrix proteins (*Col2a1, Col10a1*) and the transcription factor *Sox9* suggests that transcriptional differences are established at the cellular level, and do not simply reflect changes in overall number of cells of a given differentiation state. This pattern is supported by immunostaining against these three proteins in E15.5 tibiae, as collagens are generally weaker in both the growth plate and epiphyses in ouabain-treated tibiae (Supplementary Figures S4B,C,H). We found a similar response in gene expression after stimulating the NKA pump using monensin in tibia culture, except for differences in expression of the prechondrogenic markers *Prrx1* and *Serpina3n*, suggesting that stimulation or inhibition of the NKA pump in early embryonic tibia contributes to the regulation of mesenchymal stem cell fate during chondrogenesis. Finally, a third impact of NKA pump modulation appears to be regulation of extracellular matrix (ECM) homeostasis (Natoli et al., 2010), as NKA pump inactivation interferes with expression of collagens in both proliferative (*Col2a1*) and hypertrophic (*Col10a1*) chondrocytes (see also Supplementary Figures S4C,H), as well as proteins associated with ECM degradation (*Efemp1*, *Mmp13*).

Inferring a specific pathway or group of pathways by which NKA disruption may be inducing one or more of these changes in chondrocyte and ECM physiology is challenging; the ubiquitous NKA pump interacts with a number of pathways through ion transit, by forming complexes with membrane-bound receptors, and/or by activating a series of kinases (Therien and Blostein, 2000; Tian and Xie, 2008; Reinhard et al., 2013). Of these, we suggest two possible pathways based on prior work on the NKA pump in other tissues.

The first possibility is that the NKA pump acts directly by disrupting ion balance across the cell membrane (Cheng et al., 2019). The NKA pump forms a complex with the Na^+^–Ca^2+^ exchanger (NCX) (Tian and Xie, 2008), which can produce fluctuations in the intracellular concentration of Ca^2+^, when exposed to either ouabain (Cheng et al., 2019) or monensin (Krieger and Tashjian, 1982). These fluctuations in Ca^2+^ can facilitate localization of the transcription factor *Nf-kB* to the nucleus (Blanco and Wallace, 2013). *Nf-kB* has been shown to suppress *Sox9* expression (Rockel et al., 2008) and to suppress differentiation of MSCs into chondrocytes (Sitcheran et al., 2003). Activation of *Nf-kB* has also been shown to drive chondrocyte hypertrophy (Jimi et al., 2019) as well as osteoblast differentiation (Mishra et al., 2020). Thus, one possibility is that interfering with the NKA pump *via* ouabain or monensin increases intracellular calcium, leading to increased localization of *Nf-kB* to the nucleus, regulating gene expression in the growth plate (e.g., downregulation of *Sox9*).

A second possibility is that the NKA pump acts through modulation of a p38-MAPK signaling cascade (Feraille and Dizin, 2016). Our previous work (Stanton et al., 2004; Agoston et al., 2007) in micromass culture has shown that p38 signaling plays an important role in hypertrophic chondrocyte differentiation and regulates the expression of hypertrophic marker genes such as *Col10a1*, *Mmp13* and *Ibsp*. Here, our micromass results show that, when the NKA pump is inhibited by ouabain, *Mapk14* (p38α) expression increases in early stages of chondrocyte differentiation, with a parallel increase in expression of the ECM-related *Mmp13* that persists through later stages of differentiation (but a decrease in the expression of *Col10a1*, Figure 3).

### Altered Expression of Key Proliferative Chondrocyte Factors is Associated With Increased Bone Growth in Longshanks

Our high-throughput transcriptional analysis identified more than 400 genes with differential expression in the tibial proximal epiphysis between LS1 and Control. We conducted qPCR in tibial proximal growth plate cartilage tissue, but were able to validate only a few genes (out of 42 genes tested) as differentially expressed in LS1 compared to Control. Some differences between the results of the two methods may be due in part to differences in the tissues sampled; for RNAseq: we sampled RNA from the total epiphysis, whereas for qPCR we sampled RNA solely from the growth plate. Hence, we cannot exclude the possibility that genes differentially expressed in the bone marrow, articular cartilage, and trabecular bone, and identified in our RNAseq analysis, may play an important role in regulating bone elongation.

Our qPCR results confirm the RNASeq data showing that in both Longshanks lines three key genes are significantly differentially expressed: *Fxyd2* and *Npr3* are down-regulated*,* while *Dlk1* is upregulated. In addition, we find significant downregulation of *Bglap* and *Sox9* and upregulation of *Sfrp1* in only one of the two Longshanks lines, although a similar trend can be seen in the other (Table 1). At first glance, it is somewhat unexpected that no other major chondrogenic factors (e.g., aggrecan, collagens, Ihh, Runx2) were differentially expressed in Longshanks. One possible reason for this is that the increased length of the Longshanks tibia is associated with a larger population of proliferative cells (Marchini and Rolian, 2018). Because the deltaCT method in qPCR normalizes expression of genes of interest relative to the expression of housekeeping genes, it effectively normalizes for differences in cell number across samples. Accordingly, Longshanks may achieve longer tibiae in part by having more cells which express these specific chondrogenic factors but at similar levels to Controls.

Our cell and tissue culture data support a role for the NKA pump in chondrogenesis and/or endochondral ossification. However, the role of the NKA pump, and *Fxyd2* specifically*,* in the accelerated growth and increased length of the Longshanks tibia, is less clear. The fact that inhibition and stimulation of the NKA pump *both* reduced growth in E15.5 tibiae is at odds with increased tibia growth in Longshanks. These opposite outcomes may be because *Fxyd2* is a facultative subunit of the NKA pump that modulates its activity in a subtle, cell- and tissue-specific manner, while in our tibia culture assays ouabain and monensin exerted more potent effects on NKA activity across multiple cell types that influence bone growth, including chondrocytes, perichondral/periosteal cells, osteoblasts and/or osteoclasts.

*Npr3* (also known as *Nprc*) is a decoy receptor for C-type natriuretic peptide (CNP) (Yamashita et al., 2000) CNP is a known anabolic stimulator of bone growth that promotes proliferation of chondrocytes (Komatsu et al., 2002; Agoston et al., 2007; Teixeira et al., 2008; Esapa et al., 2016). Knockout experiments show that, in absence of *Npr3*, mice have longer bones with larger hypertrophic zones comprising both larger and more numerous chondrocytes, (Komatsu et al., 2002; Agoston et al., 2007; Teixeira et al., 2008; Esapa et al., 2016). In Longshanks growth plates, the apparently reduced presence of NPR3 protein in the proliferative zone (Supplementary Figure S2C) may increase the bioavailability of CNP, thus stimulating/maintaining chondrocyte proliferation. Npr3 may also be linked to the activation of the NKA pump but the exact activation mechanism is not entirely resolved (Moltzau et al., 2017). Interestingly, our previous work indicates that *Fxyd2* expression is downregulated in hypertrophic chondrocytes of tibiae cultured with CNP, suggesting a link between *Npr3* and *Fxyd2* expression (Agoston et al., 2007), and therefore a potential link in the downregulation of these two transcripts in both Longshanks lines.

*Dlk1*, a non-canonical Notch1 ligand that exists in both trans-membrane and soluble forms, is expressed in the proliferative and resting zones of the growth plate (Supplementary Figure S2D). *Dlk1* has been hypothesized to maintain chondrocytes in a proliferative state, and/or to delay hypertrophic maturation, likely through interactions with the Notch signaling pathway, an important regulator of cell differentiation during chondrogenesis (Mead and Yutzey, 2009a; Chen et al., 2011; Falix et al., 2012; Taipaleenmäki et al., 2012). In Longshanks, increased gene expression and immunostaining against DLK1 in chondrocytes in the resting and proliferative zone (and possibly its ECM, see Supplementary Figure S2D) is consistent with a potential role in increasing the size of the proliferative zone and thereby its growth potential (Marchini and Rolian, 2018; Gerstenfeld and Shapiro, 1996).

Lastly, we found upregulation of *Sfrp1* in LS2, and similar trends in LS1 (Henry et al., 2012). *Sfrp1* is expressed in proliferative chondrocytes and pre-hypertrophic chondrocytes of postnatal growth plates (Gaur et al., 2006) (see Supplementary Figure S2E). Sfrp1 is an antagonist of the canonical Wnt/β-catenin signaling pathway, which induces chondrocyte maturation and hypertrophy (Dong et al., 2006; Gaur et al., 2006). Although there were no overt differences in the pattern or intensity of immunostaining against SFRP1 in Longshanks and Control growth plates (Supplementary Figure S2E), we suggest that in Longshanks, increased expression of *Sfrp1* in proliferative and pre-hypertrophic chondrocytes delays their maturation and/or maintains their proliferative potential. Interestingly, upregulation of *Sfrp1* is also associated with trabecular bone deterioration (Wang et al., 2005; Yao et al., 2010; Dy et al., 2012; Marchini and Rolian, 2018). Both Longshanks lines also exhibit a phenotype of reduced trabecular ossification and bone mineral content (Farooq et al., 2017) consistent with phenotypes associated with upregulation of *Sfrp1* (Yao et al., 2010). Collectively, the altered expression of *Npr3, Dlk1, Sox9* and *Sfrp1,* points to the maintenance a larger pool of chondrocytes with proliferative potential as a key mechanism that drives the increased growth and length of the Longshanks tibia.

This study has identified a novel role of the NKA pump in controlling chondrocyte differentiation and physiology, which may contribute to variation in shape and size of skeletal elements across the body as well as intra- and interspecific variation in size and shape of individual skeletal elements. We have demonstrated that the NKA pump modulates expression of several important genes with known roles in chondrocyte proliferation, differentiation and hypertrophy, although the specific details of this mechanism remain unclear. Our results suggest several avenues for future work, which will be necessary to fully integrate the role of the NKA pump into current models of chondrocyte differentiation and transit through the growth plate.

## Data Availability

The RNA sequencing data presented in this study have been deposited in NCBI’s Gene Expression Omnibus and are accessible through GEO Series accession number GSE189528 (https://www.ncbi.nlm.nih.gov/geo/query/acc.cgi?acc=GSE189528). Additional data are provided in Supplementary Tables S2–S7.
