# Supplementary figures and images for: A Na+/K+ ATPase Pump Regulates Chondrocyte Differentiation and Bone Length Variation in Mice
Source: Front Cell Dev Biol. 2021 Dec 14;9:708384. doi: 10.3389/fcell.2021.708384 (PMC8712571; doi:10.3389/fcell.2021.708384)

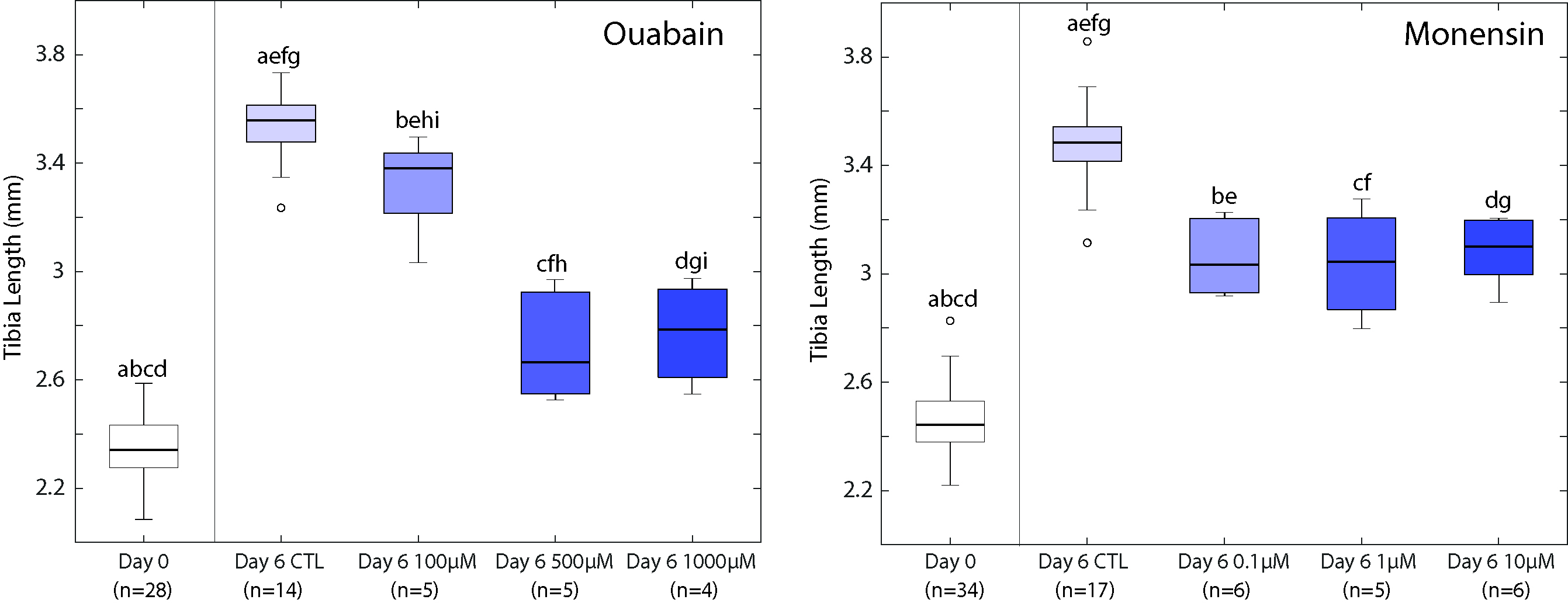

Supplement: Supplementary file 2 [file Image3.jpeg]

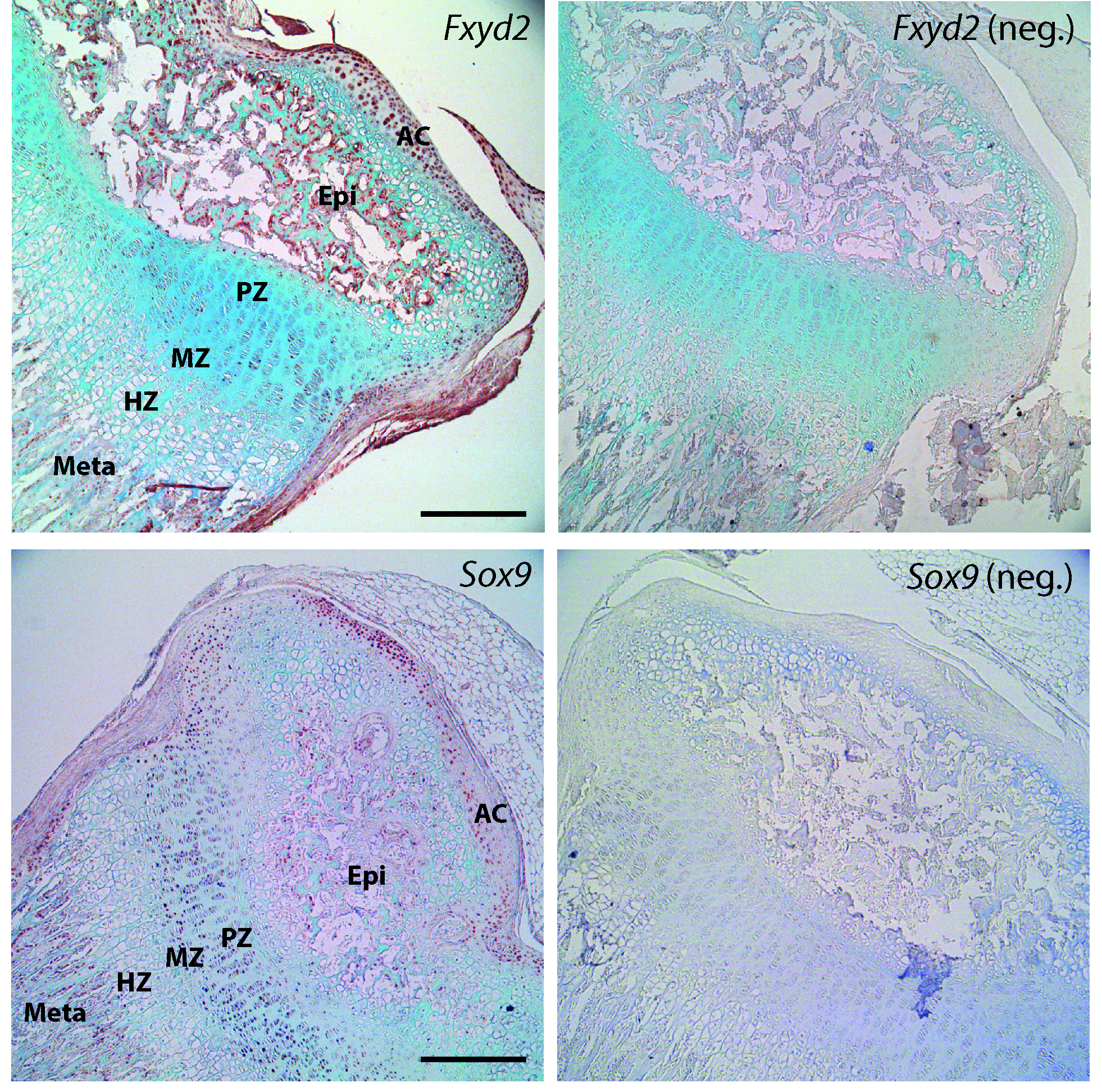

Supplement: Supplementary file 4 [file Image1.jpeg]

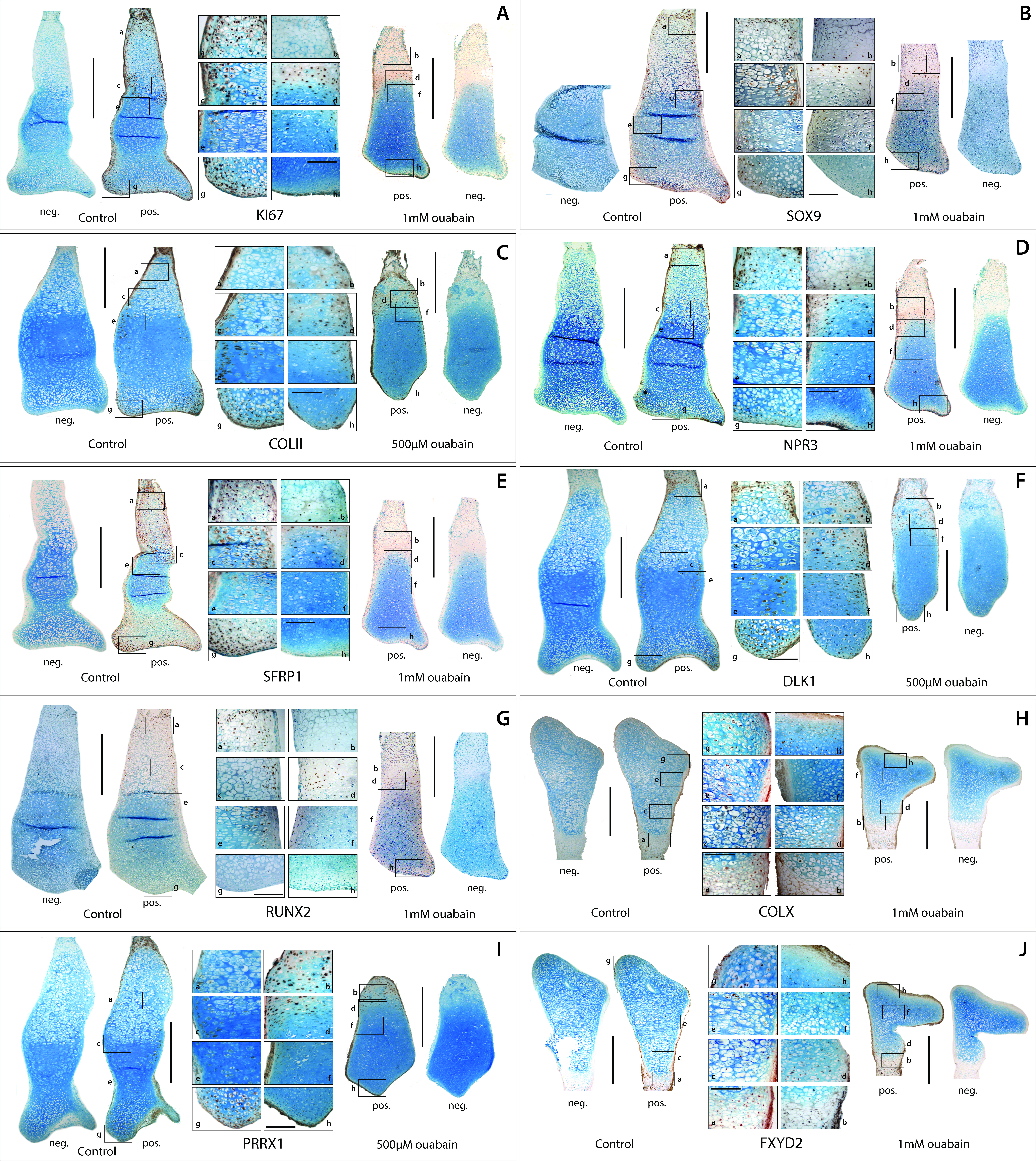

Supplement: Supplementary file 5 [file Image4.jpeg]

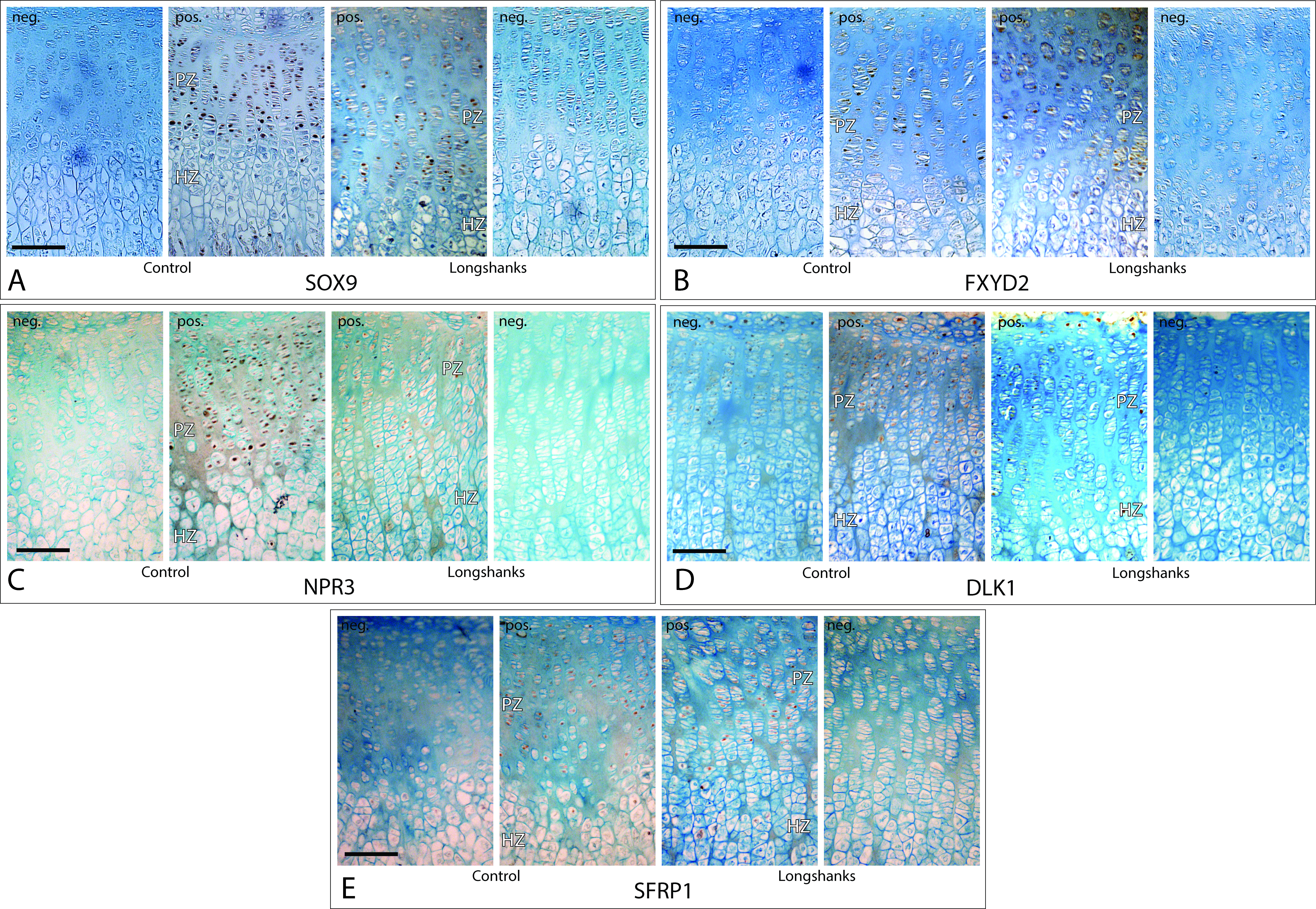

Supplement: Supplementary file 6 [file Image2.jpeg]

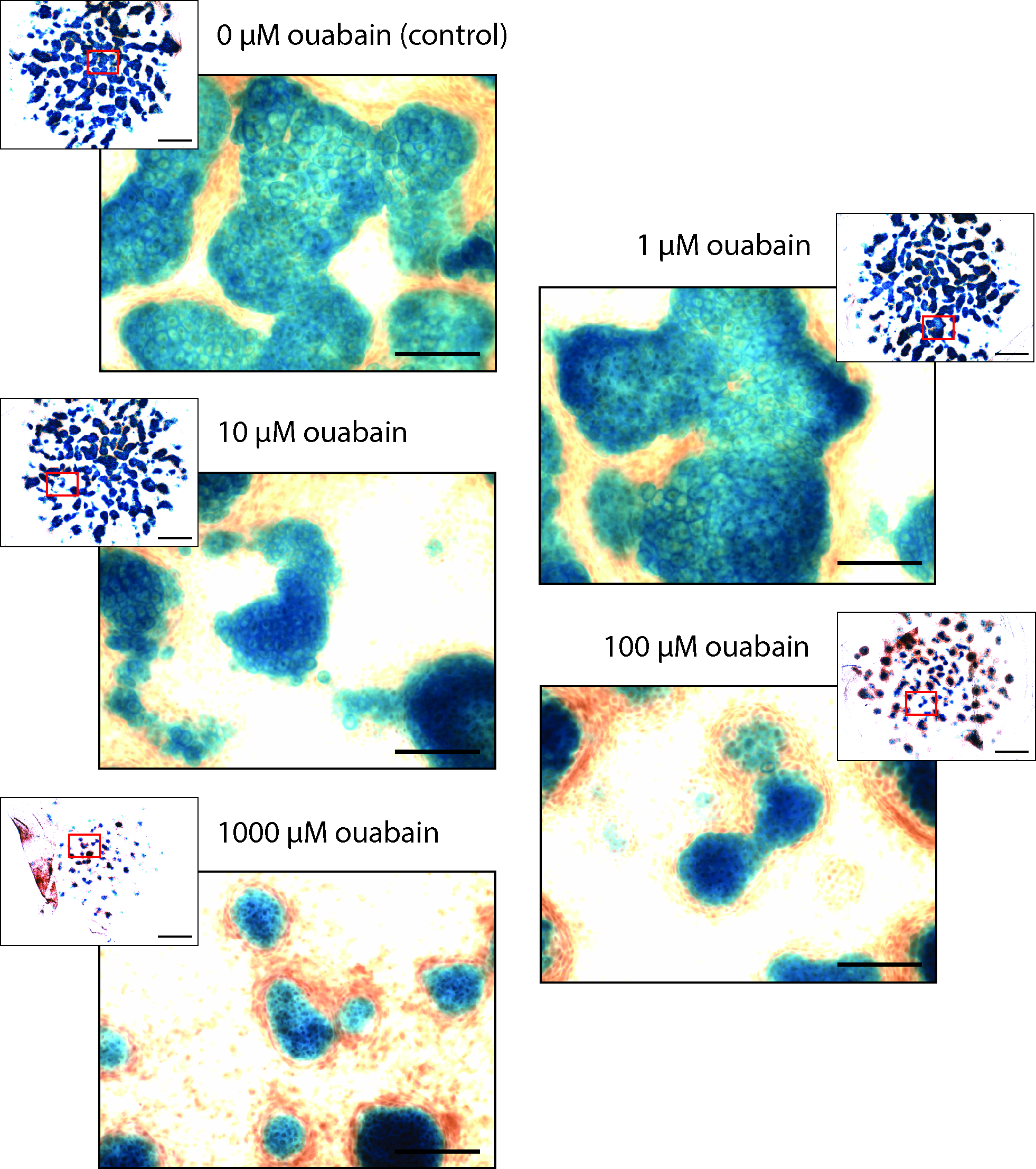

Supplement: Supplementary file 7 [file Image5.jpeg]
