# Supplementary material for: A Na+/K+ ATPase Pump Regulates Chondrocyte Differentiation and Bone Length Variation in Mice
Source: Front Cell Dev Biol. 2021 Dec 14;9:708384. doi: 10.3389/fcell.2021.708384 (PMC8712571; doi:10.3389/fcell.2021.708384)
Supplement: Supplementary file 13 [file DataSheet1.docx]

**Marchini et al. – Supplementary Information Data Sheet**

Supplementary Figures:

**Supplementary Figure S1**: Immunostaining against SOX9 (top) and FXYD2 (bottom) in proximal tibia of CD-1 mice. The distribution of FXYD2 protein, a facultative subunit of the sodium-potassium ATPase, overlaps substantially with SOX9 protein: it is strongest in articular cartilage (AC) and proliferative zone chondrocytes (PZ), but absent in the maturation or pre-hypertrophic zone (MZ) and in the hypertrophic zone (HZ). Epi = epiphysis, Meta = metaphysis. Negative control sections (neg.) were incubated with normal serum without primary antibody. Scale bar: 200µm.

**Supplementary Figure S2**: Immunostaining against SOX9 (A), FXYD2 (B), NPR3 (C), DLK1 (D), and SFRP1 (E) in postnatal day 14 proximal tibia growth plates from Control and Longshanks 1. PZ = proliferation zone, HZ = hypertrophic zone. Negative control sections (neg.) were incubated with normal serum without primary antibody. Scale bar: 100µm.

**Supplementary Figure S3:** Embryonic tibia growth over six days of culture in serial dilutions of ouabain (left) or monensin (right). Black lines indicate medians, boxes represent interquartile ranges, bars represent non-outlier ranges and open circles show outliers. Superscript letters indicate significant differences in the means of pairs of treatments, as determined by ANOVA followed by post-hoc pairwise Tukey’s tests. Sample sizes for ouabain: Day 0 Controls (n=28), Day 6 Controls (n=14), Day 6 100µM (n=5), Day 6 500µM (n=5), Day 6 1000µM (n=4). Sample sizes for monensin: Day 0 Controls (n=34), Day 6 Controls (n=17), Day 6 0.1µM (n=6), Day 6 1µM (n=5), Day 6 10µM (n=6).

**Supplementary Figure S4**: Immunostaining against selected chondrocyte proliferation, hypertrophy and differentiation factors in growth plates and epiphyses (distal, unless otherwise noted) from E15.5 embryonic tibiae grown in normal media (left) vs media supplemented with ouabain (right) (1000µM, unless otherwise noted). A: KI67; B: SOX9, C: COLII (500µM ouabain), D: NPR3; E: SFRP1; F: DLK1 (500µM ouabain) G: RUNX2; H: COLX (proximal epiphysis); I: PRRX1 (500µM ouabain); J: FXYD2 (proximal epiphysis). Insets show magnification of regions approximating the hypertrophic zone (a,b), maturation/pre-hypertrophic zone (c,d), proliferative zone (e,f), and articular cartilage (g,h). . Negative control sections (neg.) were incubated with normal serum without primary antibody. Full view scale bar: 500µm ; Inset scale bar: 100µm

**Supplementary Figure S5**: Alcian blue/Alizarin red staining of micromasses cultured in serial dilutions of ouabain octahydrate for 9 days (day 6 to day 15). Smaller images are lower magnification (10x) views showing the overall appearance of a typical micromass for each ouabain concentration. Note the greater confluence of blue-stained cartilage nodules in lower doses of ouabain and control media, and the decreasing size of the nodules and increased presence of calcified matrix (stained red) in higher concentrations of ouabain. Scale bar = 1mm. Insets show 40x magnified regions outlined in the red boxes in the low magnification views. Inset scale bar = 100µm

Supplementary Tables:

**Supplementary Table S1**: TaqMan assay catalog numbers

**Supplementary Table S2**: HiSat2 DEseq analysis of RNA sequencing data

**Supplementary Table S3**: Longshanks growth plate deltaCT values

**Supplementary Table S4**: Tibia culture bone lengths

**Supplementary Table S5**: Ouabain tibia culture deltaCT values

**Supplementary Table S6**: Monensin tibia culture deltaCT values

**Supplementary Table S7**: Micromass culture deltaCT values
